# Titanium Nitride Nanodonuts Synthesized from Natural Ilmenite Ore as a Novel and Efficient Thermoplasmonic Material

**DOI:** 10.3390/nano11010076

**Published:** 2020-12-31

**Authors:** Thanh-Lieu Thi Le, Lam Tan Nguyen, Hoai-Hue Nguyen, Nguyen Van Nghia, Nguyen Minh Vuong, Hoang Nhat Hieu, Nguyen Van Thang, Viet Thong Le, Viet Huong Nguyen, Pin-Cheng Lin, Anupam Yadav, Ivan Madarevic, Ewald Janssens, Hao Van Bui, Loan Le Thi Ngoc

**Affiliations:** 1 Faculty of Natural Sciences, Quy Nhon University, 170 An Duong Vuong, Quy Nhon City 590000, Vietnam; lethithanhlieu@qnu.edu.vn (T.-L.T.L.); nguyentanlam@qnu.edu.vn (L.T.N.); nguyenvannghia@qnu.edu.vn (N.V.N.); nguyenminhvuong@qnu.edu.vn (N.M.V.); hoangnhathieu@qnu.edu.vn (H.N.H.); nguyenvanthang@qnu.edu.vn (N.V.T.); 2 Faculty of Electrical and Electronic Engineering, Phenikaa University, Yen Nghia Ward, Ha Dong District, Hanoi 12116, Vietnam; hue.nguyenhoai@phenikaa-uni.edu.vn (H.-H.N.); thong.leviet@phenikaa-uni.edu.vn (V.T.L.); huong.nguyenviet@phenikaa-uni.edu.vn (V.H.N.); 3 Faculty of Materials Science and Engineering, Phenikaa University, Yen Nghia Ward, Ha Dong District, Hanoi 12116, Vietnam; 4 Quantum Solid-State Physics, Department of Physics and Astronomy, KU Leuven, 3001 Leuven, Belgium; pincheng.lin@kuleuven.be (P.-C.L.); anupam.yadav@kuleuven.be (A.Y.); ivan.madarevic@kuleuven.be (I.M.); ewald.janssens@kuleuven.be (E.J.)

**Keywords:** titanium nitride, thermoplasmonic, nanodonuts, water evaporation

## Abstract

Nanostructures of titanium nitride (TiN) have recently been considered as a new class of plasmonic materials that have been utilized in many solar energy applications. This work presents the synthesis of a novel nanostructure of TiN that has a nanodonut shape from natural ilmenite ore using a low-cost and bulk method. The TiN nanodonuts exhibit strong and spectrally broad localized surface plasmon resonance absorption in the visible region centered at 560 nm, which is well suited for thermoplasmonic applications as a nanoscale heat source. The heat generation is investigated by water evaporation experiments under simulated solar light, demonstrating excellent solar light harvesting performance of the nanodonut structure.

## 1. Introduction

The emerging field of thermoplasmonics uses metal nanoparticles (NPs) and metal metamaterial structures as nanoscale heat sources when excited at their localized plasmon resonance wavelength through incident light absorption [1,2]. This has been employed for various applications, such as cancer therapy, photothermal imaging, photothermal and hot-electron enhanced chemistry, and applications based on solar light harvesting [3].

Solar light is a very important source of environmentally clean and sustainable energy. Thermoplasmonic systems are particularly interesting for solar light harvesting applications, such as thermophotovoltaics and solar water evaporation (SWE). Since ancient times, SWE has been a fundamental technology for potable water production [4,5]. This technology has gained even more attention nowadays due to its great potential for addressing global challenges, such as clean water shortage (e.g., people in remote areas during the flooding season, fisherman on an unexpected long trip on the sea), desalination, and wastewater treatment [6,7,8,9]. Generally, in SWE, sunlight is absorbed by a photothermal material (i.e., absorber), which is converted into heat to vaporize water [10]. Due to their broad absorption range, carbon nanomaterials, such as amorphous carbon, graphene, and carbon nanotubes, are high-efficiency solar light absorbers. Although their low emissivity is a limiting factor for achieving high-efficiency photothermal conversion, various carbon-based materials and structures have demonstrated good SWE performance [9,11,12,13,14].

Pioneering research utilizing thermoplasmonics for SWE employed solar harvesting with metal NPs dispersed in a liquid [15,16,17]. Typically, noble metal NPs, such as Au, Ag, Pt, and Pd, have been employed because of their widespread use in plasmonics and strong light absorption at the localized surface plasmon resonance [10,15,17]. However, their high cost and narrow absorption range are hindrances for practical SWE deployment. Recently, titanium nitride (TiN) has been demonstrated as a highly stable plasmonic material that is much cheaper than noble metals [18,19,20,21,22,23,24,25]. TiN NPs have been reported to be promising for solar harvesting applications, in which efficient nanoscale heat generators with a wide spectral absorption range are highly desirable [21,26,27]. Employing these advantages, TiN has been demonstrated as an excellent photothermal material for SWE [21,22,23,28,29,30]. Furthermore, since evaporation occurs at the liquid–air interface, and heat is generated in the bulk liquid, the volumetric heating method usually achieves low energy conversion efficiency due to the heat loss [5,28]. Therefore, recent SWE studies have employed floating structures, in which the photothermal material is immobilized on a substrate that floats in water. Using this approach, significant improvements of the SWE efficiency have been achieved [9,11,14,30,31,32].

In this work, we present a low-cost method for fabricating a novel nanostructure of TiN, i.e., nanodonut. The TiN nanodonuts exhibit strong and spectrally broad localized surface plasmon resonance absorption in the visible region that provides excellent photothermal conversion performance. We demonstrate the effectiveness of the TiN nanodonuts as broad-band thermoplasmonic heat generators for SWE under simulated solar light using the floating substrate approach by depositing the TiN nanodonuts on a polymer membrane.

## 2. Experimental Section

### 2.1. Materials and Chemicals

Ammonium hydroxide (NH_4_OH, 25%), hydrofluoric acid (HF, 48%), potassium chloride (KCl, 99%), and carbon nanopowders (average diameter of 100 nm) were purchased from Sigma-Aldrich Co., Ltd. (St. Louis, MO, USA). Ilmenite ore with the chemical composition 49.5% TiO_2_, 32.7% Fe_2_O_3_, 11.2% FeO, 0.2% SiO_2_ and 6.4% other impurities was provided by Binh Dinh Minerals Joint Stock Co., Vietnam.

### 2.2. Synthesis of TiO_2_ Nanoparticles from Ilmenite Ore

TiO_2_ NPs were synthesized by a three-step process described as follows:

Step 1: Ilmenite ore was firstly crushed and ground into fine powder with particle sizes in the range of 50–75 µm. Then, 10 g of the powder was transferred into a 250 mL plastic beaker containing 70 mL of HF 20% solution. The suspension was continuously stirred for 5 h at room temperature. The obtained slurry suspension (i.e., filtrate) was separated from the deposited solid residual.

Step 2: 30 mL KCl 4 M solution was slowly added to the filtrate, resulting in a white K_2_TiF_6_ precipitate. In the next step, the precipitate was separated and dissolved in water by heating up the suspension to 80 °C until a saturated solution was achieved, which was then filtered and rapidly cooled down to room temperature to form again K_2_TiF_6_ precipitate. This step was used to eliminate the impurities and purify the K_2_TiF_6_ precipitate. The precipitate was dried in air at 105 °C for 2 h.

Step 3: 5 g of K_2_TiF_6_ precipitate was dissolved in 500 mL of distilled water by heating up to 80 °C. Then, NH_3_ solution (4 M, prepared from ammonium hydroxide 28% solution) was slowly added until pH = 9. This hydrolysis reaction produced Ti(OH)_4_, which was then annealed at 550 °C for 3 h to obtain TiO_2_.

### 2.3. Synthesis of TiN by Nitridation of TiO_2_ in NH_3_

Nitridation of TiO_2_ to obtain TiN has been reported by several research groups [33,34,35,36,37]. In our approach, for each experiment, 1 g of TiO_2_ NPs was loaded into a ceramic boat and placed at the center of a quartz-tube furnace (PTF 12/50/610, Lenton, UK). One end of the tube was connected to the gas inlet (N_2_, NH_3_). The other end was connected to a mechanical vacuum pump. Initially, the quartz tube was evacuated to reach a vacuum of 10^−2^ mbar and then pre-heated to 250 °C. The tube was purged several times by N_2_ (99.99%) to remove contaminants. Thereafter, the temperature in the furnace was increased to either 700 or 900 °C, both at a ramping rate of 3 °C min^−1^. After the temperature was stabilized, NH_3_ gas was introduced into the furnace at a flow rate of 1000 sccm for 1 h. Finally, the furnace was cooled down to 100 °C in NH_3_ ambient, and further to room temperature in N_2_ before unloading the sample.

### 2.4. Material Characterizations

The morphology of the materials was studied by Field-Emission Scanning Electron Microscopy (FE-SEM) and High-Resolution Transmission Electron Microscopy (HR-TEM) using Hitachi S4800 (Ibaraki, Japan) and JEOL ARM-200F (Tokyo, Japan) systems, respectively. The crystalline structure of the materials was investigated by X-Ray Diffraction (XRD) using a Bruker diffractometer (D8 Advance Eco, Bruker, Billerica, MA, USA) equipped with a Cu Kα X-ray radiation source. The optical absorption spectra were acquired by using a JASCO V-750 UV–VIS spectrophotometer (Easton, MD, USA). Chemical compositions of the materials and the binding energy of the elements were determined by X-Ray Photoelectron Spectroscopy (XPS) using a XR4 Thermo Scientific Spectrometer (Waltham, MA, USA) equipped with an Mg-Kα X-ray radiation source.

### 2.5. Solar Water Evaporation Experiments

For each experiment, 20 mg of the powder (TiO_2_, TiON, TiN or nanocarbon) was dispersed in ethanol and sonicated for 10 min. Using the drop-coating method, the powder was deposited on a polymer membrane (Novatexx 2471, Freudenberg, 5 cm in diameter). The membrane was immersed in water contained in a 100 mL glass beaker and was kept afloat at a distance of ~5 mm below the water surface, which was the equilibrium position of the membrane when it floated. It is worth noting that due to the non-uniform mass distribution, the membrane might be slightly tilted. To address this issue, a thin fabric string was used to keep the entire membrane in the horizontal position. The evaporation was investigated by monitoring the weight change of the system (glass beaker, water and the membrane) under simulated solar light generated by a Xenon arc lamp (60 W, Guangzhou Lightech Auto Lighting Co., Ltd. Guangdong, China) with an illuminance of 550 W m^−2^, which is equivalent to an illuminance of 0.55 sun of natural solar light. The temperatures of the environment, the surface of the membranes and the liquid were measured using a BETEX 1230 Infrared Thermometer (Bega Special Tools, Vaassen, the Netherlands).

## 3. Results and Discussion

**Figure 1:**
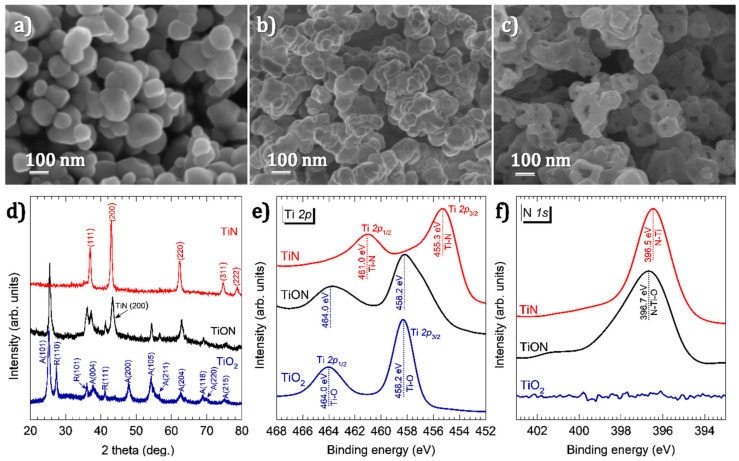
SEM images of (**a**) the TiO_2_ powder obtained directly from the hydrofluoric acid leaching process and (**b**) after annealing the powder in NH_3_ at 700 °C and (**c**) after annealing at 900 °C; (**d**) XRD patterns of the powders in images (**a**–**c**), labelled as TiO_2_, TiON and TiN, respectively; XPS spectra of TiO_2_, TiON and TiN (**e**) at the Ti 2p binding energy and (**f**) at the N 1s binding energy.

**Figure 2:**
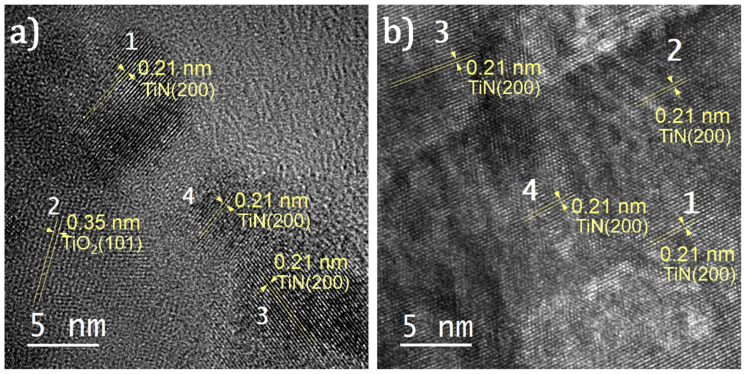
HR-TEM images of (**a**) TiON and (**b**) TiN. The lattice spacing was measured using Gatan Micrograph Suite^®^ software. The measured lattice spacing of 0.21 nm corresponds to the spacing between (200) planes of TiN, whereas the lattice spacing of 0.35 nm corresponds to the spacing between the (101) planes of anatase TiO_2_ [41].

The hydrofluoric acid leaching of ilmenite ore produces TiO_2_ NPs with sizes in the range of 70–160 nm, as shown in the SEM micrograph in Figure 1a. The presence of TiO_2_ material is confirmed by XRD and XPS analyses shown in Figure 1d and Figure 1e, respectively. The XRD pattern from the obtained powder (Figure 1d, bottom pattern) is consistent with that of the polycrystalline TiO_2_ containing both anatase and rutile phases [38]. The XPS spectrum of the Ti 2p core-level (Figure 1e, bottom spectrum) shows two peaks at binding energies of 464.0 and 458.2 eV. These peaks are the characteristic doublet state of Ti 2p (i.e., Ti 2p_1/2_ and Ti 2p_3/2_, respectively) in TiO_2_ [39]. Following annealing in NH_3_ at 700 °C for 1 h, a slight coalescence of the NPs is observed (Figure 1b). The annealing strongly affects the crystalline structure and chemical composition, as shown in the spectra in Figure 1d–f. In the XRD pattern (Figure 1d), the R(110) and A(200) peaks observed for TiO_2_ vanish and a new peak at 43.3° appears. This peak represents the (200) plane of TiN cubic structure [40], and is further confirmed by the HR-TEM image shown in Figure 2a. The co-existence of both TiO_2_ and TiN results in the N–Ti–O bonds, causing the broadening of the Ti 2p peaks to the lower binding energy side, as shown in Figure 1e (middle spectrum) [39]. The presence of those bonds is also demonstrated by the broad and asymmetric N 1s peak shown in Figure 1f (middle spectrum) [39]. The XRD and XPS analyses indicate that the nitridation of the TiO_2_ at 700 °C was incomplete, resulting in TiO_2_–TiN composite (hereafter denoted as TiON).

After nitriding at 900 °C in NH_3_ for 1 h, the NPs exhibit distinct changes in morphology: the NPs transform into an entirely different structure with the shapes of nanodonuts having outer diameters in the range of 80–120 nm, and inner diameters ranging from 30 to 60 nm (Figure 1c and Figure S1,). In the XRD pattern shown in Figure 1d, the diffraction peaks of TiO_2_ entirely disappear, and only TiN peaks are observed [27]. This is further supported by the results obtained by HR-TEM shown in Figure 2b. Furthermore, the two peaks at 461.0 and 455.3 eV in the Ti 2p XPS spectrum (Figure 1e) and the peak at 396.5 eV in the N 1s spectrum (Figure 1f) are consistent with the binding energies of Ti–N bonds in stoichiometric TiN [39]. Therefore, we conclude that by annealing in NH_3_ at 900 °C, the TiO_2_ was completely nitridized and transformed into TiN.

The nitridation of TiO_2_ in NH_3_ ambient was previously explained by Gou et al. [42]. According to the study, at 900 °C, the nitridation takes place via the formation of TiN_1−x_O_x_ and releases H_2_O vapor and N_2_ gas. With increasing the reaction time, the oxygen atoms of TiN_1−x_O_x_ are gradually substituted by the nitrogen atoms. Eventually, TiO_2_ is nitridized to TiN [42]. Importantly, the authors observed the formation of mesopores with diameters in the range of 20–40 nm after the nitridation. This is consistent with formation of the cavities, which results in the nanodonuts; this can be attributed to the release of H_2_O vapor and N_2_ gas. In addition, it has been reported that the incorporation of nitrogen atoms during the nitridation process can cause an expansion and contraction of the particles [43,44], which can be another factor that promotes the formation of the nanodonut structure. Nevertheless, this assumption requires further studies for clarification.

**Figure 3:**
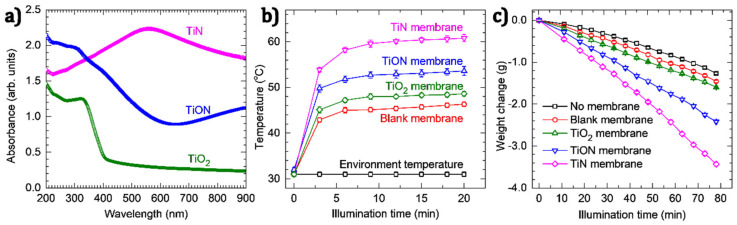
(**a**) UV–VIS absorption spectra of TiO_2_, TiON and TiN nanodonuts, (**b**) temperature at the surface of membranes under simulated solar light with illuminance of 550 W m^–2^, and (**c**) the weight change due to water evaporation by different photothermal materials.

The UV–VIS diffuse reflectance spectra of the materials are shown in Figure 3a. The TiO_2_ NPs have an absorption edge at 410 nm, which corresponds to a bandgap of 3.0 eV (using the Tauc method). The absorption of the TiON NPs exhibits a significant red shift that results in a bandgap of 2.1 eV. The TiN nanodonuts manifest a broad plasmon resonance spectrum in the visible region with a peak centered at 560 nm. This is in contrast to resonance peaks commonly observed for TiN, which are in the far-red and near infrared ranges. For instance, Traver et al. reported a peak plasmon resonance at 760 nm for TiN NPs with diameters of 10–20 nm [23]; Ishii et al. reported a peak at 700 nm for TiN NPs with sizes of about 30 nm [27]; whereas Hao et al. reported a peak in the NIR region for the TiN NPs with diameters of 80–90 nm [40]. These examples demonstrate a non-monotonic relationship between the particle size and the plasmon resonance of TiN NPs. We speculate that the peak resonance at 560 nm, in this case, may arise from the structure of the nanodonut NPs, which requires further exploration. Importantly, the broad plasmon resonance spectrum of the TiN nanodonuts corresponds well with the solar spectral range where sunlight provides the highest flux (Figure S2, Supporting Information). This is highly desirable for the solar light harvesting applications.

Figure 3b shows the measured temperatures of the polymer membrane coated with TiO_2_, TiON and TiN nanodonuts (hereafter denoted as TiO_2_, TiON and TiN membranes, respectively) under continuous-wave (cw) illumination of simulated solar light generated by a Xenon arc lamp with illuminance of 550 W m^–2^. The measurements were performed in air at a relative humidity of 72% and an ambient temperature of 31 °C. The results demonstrate that, after 9 min of cw illumination, the temperature of the blank membrane increases from 31 to 45 °C and stabilizes thereafter. Higher temperatures are acquired for the TiO_2_ and TiON membranes (i.e., 48 and 53 °C, respectively), which can be explained by the improved light absorption (Figure 3a). For the TiN membrane, the temperature reaches 60 °C, indicating its higher photothermal conversion efficiency.

The use of TiN nanodonuts as nanoscale heat generators was tested by studying their SWE performance under cw illumination of simulated solar light with illuminance of 550 W m^−2^. For this purpose, the membranes were immersed in water and kept at a position of about 5 mm below the water surface. Water evaporation was investigated by monitoring the weight change under continuous cw simulated solar illumination. The results are shown in Figure 3c, demonstrating a linear decrease in weight after 10 min of cw illumination. From these plots, the evaporation rates are calculated, which are presented in Table S1, Supporting Information. For the TiN membrane, an evaporation rate of 0.045 g min^−1^ is achieved. Taking into account the diameter of the glass beaker (~5 cm) gives an evaporation rate of 1.38 kg h^−1^ m^−2^. This rate is comparable to evaporation rates obtained for various other materials, which are typically in the range of 1.0–1.9 kg h^−1^ m^−2^, despite our lower illuminance (Table 1). This suggests the high light harvesting efficiency of the TiN nanodonuts. In addition, the TiN nanodonuts outperform carbon and graphene NPs under similar experimental conditions (Table S1 and Figure S3, Supporting Information). Furthermore, it is worth mentioning that the TiN membrane was used for a considerable number of experiments (i.e., above 30) in various experimental conditions (e.g., under simulated solar light, under natural solar light, in fresh water and in salt water with a concentration of 35 g L^−1^) with total illumination time above 30 h. The data reported in Figure 3c were acquired after the membrane had been used for more than 25 h. No considerable change in the evaporation rate (as well as the formation of air bubbles presented in the next part) was observed. This indicates an excellent stability of the TiN nanodonuts.

**Figure 4:**
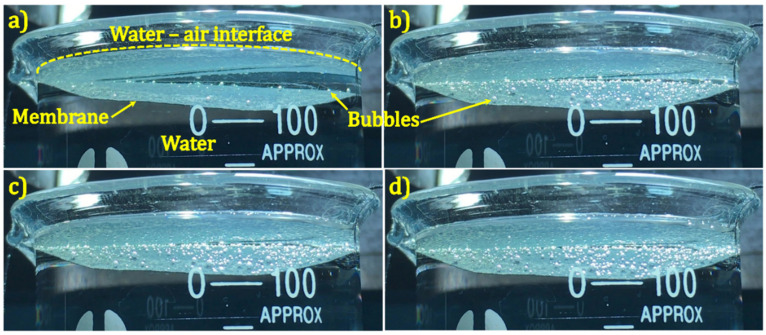
Bubbles generated on TiN membrane surface under continuous illumination for (**a**) 30 s, (**b**) 600 s, (**c**) 1200 s and (**d**) 1800 s. The membrane is slightly tilted for better observation.

Importantly, we observed that within 30 s of illumination, bubbles were formed at the TiN membrane surface (Figure 4). Under continuous cw illumination, the bubbles expanded their volume and eventually detached from the membrane surface and moved to the water–air interface, where the air contained in the bubbles was released (Videos S1 and S2, Supporting Information). Only sporadic bubbles were observed for the TiON membrane and no bubble was observed for the blank and the TiO_2_ membranes (Figure S4, Supporting Information). This can be explained by the higher temperature of the TiN membrane (i.e., 60 °C), as shown in Figure 3b. The bubble formation due to the thermoplasmonic effect has been described in detail by Baffou et al. [2,52]. Two important conclusions emerge from their analysis: (i) the bubbles contain air, and (ii) the NPs generate a high localized temperature in the range of 200–220 °C, which is required to initiate bubble generation [2,3,52]. From their second conclusion, the bubble formation observed in our work suggests that the local temperature obtained for the TiN nanodonuts under cw illumination could be significantly higher than the measured value at the surface of the TiN membrane (i.e., 60 °C). This seeming discrepancy can be attributed to the fact that the infrared temperature probe has a spot size of about 2 mm and thus provides a spatially averaged value, while the bubble formation occurs locally. We note that bubble formation caused by the thermoplasmonic effect has been observed for Au NPs by many research groups [2,15,17,53]. However, this phenomenon has not been reported for TiN, although high local heat has been suggested for various TiN nanostructures under simulated solar light illumination [19,21,23,27,28,29].

## 4. Conclusions

In summary, we have demonstrated a low-cost and feasible approach for the fabrication of TiN nanodonuts that exhibit strong and broad plasmon resonance absorption in the visible region centered at 560 nm. The SWE performance was studied using a floating structure prepared by drop-coating the TiN nanodonuts on a polymer membrane. Using simulated solar light with an illuminance of 550 W m^−2^, our experiments reveal two important observations. First, the TiN nanodonuts provide an evaporation rate of 1.38 kg h^−1^ m^−2^. This value is comparable to previously reported rates obtained for higher illuminance, proving that the TiN nanodonuts are highly efficient light harvesting materials. Second, the formation of the bubbles at the membrane surface is observed, providing firm evidence of high local heat generated by the TiN nanodonuts, which has not been previously reported.

## Figures and Tables

**Table 1:** Solar water evaporation (SWE) performance of various photothermal materials reported in the literature.

Photothermal Material	Floating Substrate	Light Intensity (kW m^−2^)	Evaporation Rate (kg h^−1^ m^−2^)	Reference
TiN nanodonuts	Polymer membrane	0.55	1.38	This work
TiN NPs	Ceramic fiber wool	1.0	1.847	[21]
TiN NPs	Mesoporous anodized alumina membrane	1.21	1.606	[29]
Ti_2_O_3_ NPs	Cellulose membrane	1.0	1.32	[45]
RGO-Sodium alginate-CNT aerogel	Self-floating	1.0	1.622	[13]
2D GO film	Cellulose-wrapped Polystyrene foam	1.0	1.45	[46]
Carbon black coated PMMA nanofiber on PAN nanofiber	Self-floating	1.0	1.3	[47]
Bi-layered rGO film	Polystyrene foam	1.0	1.31	[48]
Carbon nanotubes	Porous Silica	1.0	1.32	[49]
Flame-treated wood	Self-floating	1.0	1.05	[50]
Carbonized mushrooms	Polystyrene foam	1.0	1.475	[51]

## Data Availability

The data presented in this study are available on request from the corresponding author.

## Supplementary Materials

The following are available online at https://www.mdpi.com/2079-4991/11/1/76/s1, Figure S1: Representative TEM images of TiN nanodonuts. The particle and cavity sizes were measured using Gatan Micrograph Suite^®^ software, Figure S2: UV-Vis absorption spectrum of TiN nanodonuts and solar emission spectrum, Figure S3: SWE performance of the synthesized photothermal materials (TiO_2_, TiON and TiN) in comparison with the graphene nanoplatelets and carbon nanopowders, Figure S4: Photographs of (a) blank polymer membrane, (b) TiO_2_ membrane, (c) TiON membrane and (d) TiN membrane taken after 600 s of exposure to simulated solar light generated by the Xenon arc lamp with an illuminance of 550 W m^–2^, Table S1: Evaporation rate of water without the membranes, the blank membrane and the membranes deposited with photothermal nanomaterials (TiO_2_, TiON, TiN, carbon and graphene NPs) under simulated solar light with an illuminance of 550 W m^–2^, Video S1: Formation of bubbles on TiN membrane under continuous illumination of simulated solar light with an illumination of 550 W m^−2^, Video S2: Evaporation of water in the presence of the TiN membrane under continuous illumination of simulated solar light with an illumination of 550 W m^−2^.
