# Supplementary material for: Titanium Nitride Nanodonuts Synthesized from Natural Ilmenite Ore as a Novel and Efficient Thermoplasmonic Material
Source: Nanomaterials (Basel). 2020 Dec 31;11(1):76. doi: 10.3390/nano11010076 (PMC7823350; doi:10.3390/nano11010076)
Supplement: Supplementary file 1 [file nanomaterials-11-00076-s001.zip › nanomaterials-1054750 - supplementary xml pdf.pdf]

nanomaterials

Supporting Information

Titanium Nitride Nanodonuts Synthesized from Natural Ilmenite Ore as a Novel and Efficient Thermoplasmonic Material

Thanh-Lieu Thi Le [1], Lam Tan Nguyen [1], Hoai-Hue Nguyen [2], Nguyen Van Nghia [1], Nguyen Minh Vuong [1], Hoang Nhat Hieu [1], Nguyen Van Thang [1], Viet Thong Le [2,3], Viet Huong Nguyen [2,3], Pin-Cheng Lin [4], Anupam Yadav [4], Ivan Madarevic [4], Ewald Janssens [4], Hao Van Bui [2,3,*] and Loan Le Thi Ngoc [1,*]

[1] Faculty of Natural Sciences, Quy Nhon University, 170 An Duong Vuong, Quy Nhon City 590000, Vietnam; lethithanhlieu@qnu.edu.vn (T.-L.T.L.); nguyentanlam@qnu.edu.vn (L.T.N.); nguyenvannghia@qnu.edu.vn (N.V.N.); nguyenminhvuong@qnu.edu.vn (N.M.V.); hoangnhathieu@qnu.edu.vn (H.N.H.); nguyenvanthang@qnu.edu.vn (N.V.T.)

[2] Faculty of Electrical and Electronic Engineering, Phenikaa University, Yen Nghia Ward, Ha Dong District, Hanoi 12116, Vietnam; hue.nguyenhoai@phenikaa-uni.edu.vn (H.-H.N.); thong.leviet@phenikaa-uni.edu.vn (V.T.L.); huong.nguyenviet@phenikaa-uni.edu.vn (V.H.N.)

[3] Faculty of Materials Science and Engineering, Phenikaa University, Yen Nghia Ward, Ha Dong District, Hanoi 12116, Vietnam

[4] Quantum Solid-State Physics, Department of Physics and Astronomy, KU Leuven, 3001 Leuven, Belgium; pincheng.lin@kuleuven.be (P.-C.L.); anupam.yadav@kuleuven.be (A.Y.); ivan.madarevic@kuleuven.be (I.M.); ewald.janssens@kuleuven.be (E.J.)

* Correspondence: hao.buivan@phenikaa-uni.edu.vn (H.V.B.); lethingocloan@qnu.edu.vn (L.L.T.N.); Tel.: +84-961-960-639 (L.L.T.N.)

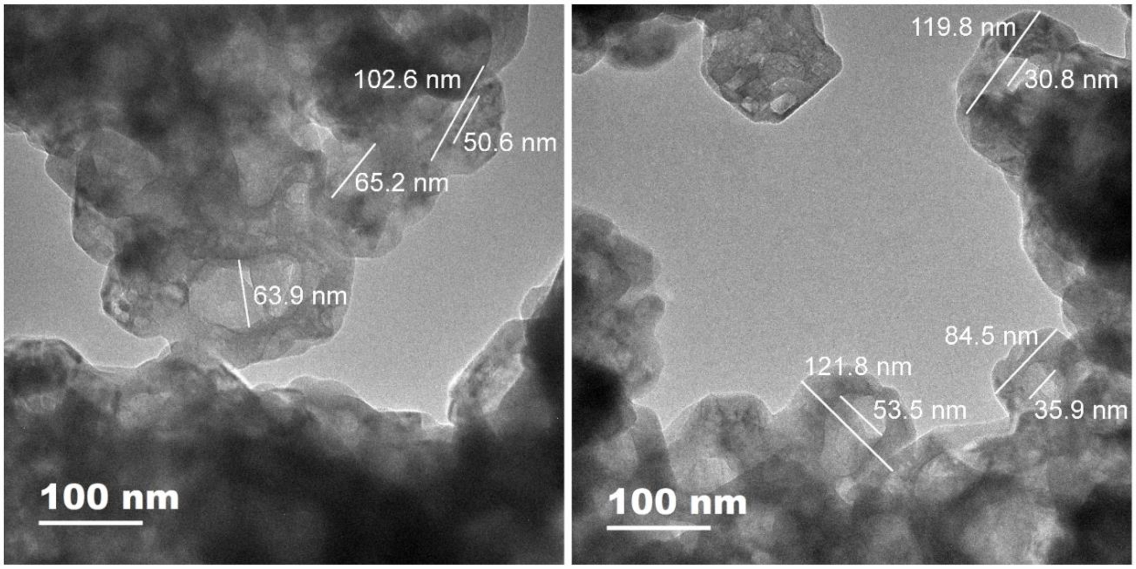

Figure S1. Representative TEM images of TiN nanodonuts. The particle and cavity sizes were measured using Gatan Micrograph Suite® software.

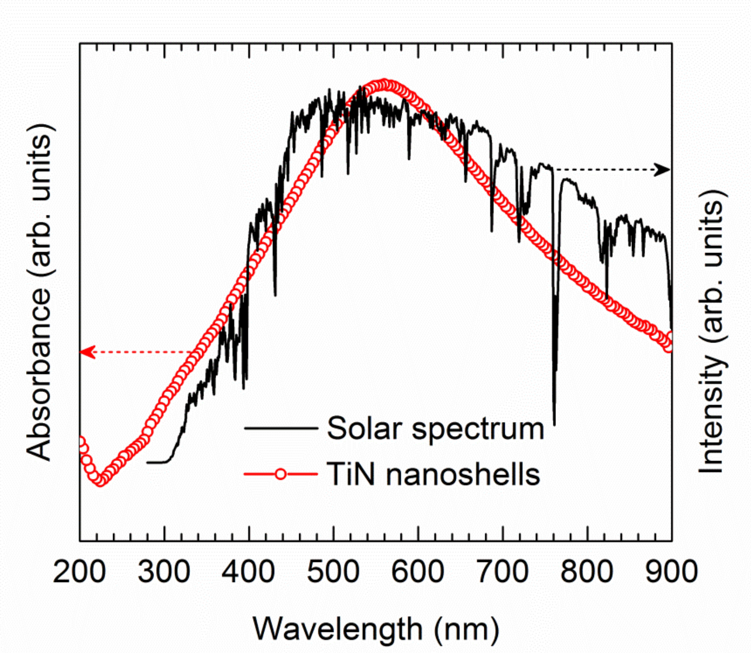

Figure S2. UV-Vis absorption spectrum of TiN nanodonuts and solar emission spectrum.

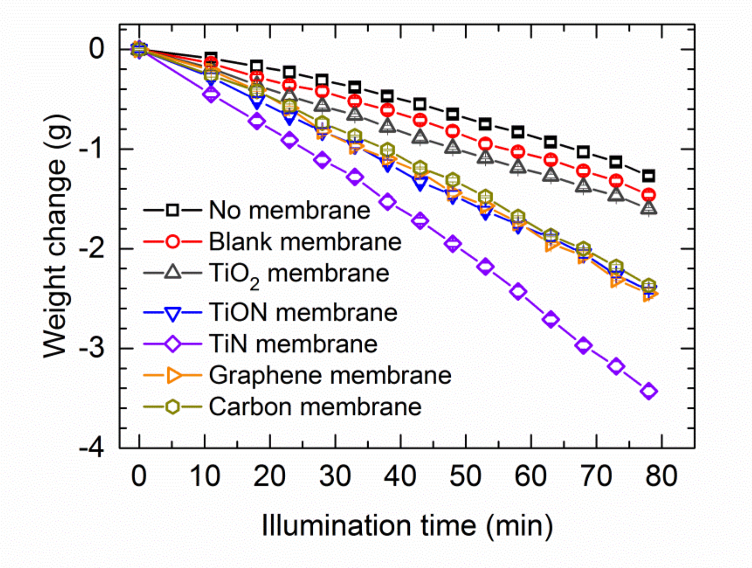

Figure S3. SWE performance of the synthesized photothermal materials (TiO_2, TiON and TiN) in comparison with the graphene nanoplatelets and carbon nanopowders. For all of the measurements, the same amount of material (i.e., 20 mg) was deposited on the polymer membranes with the same size (5 cm in diameter) by drop-coating method.

Table S1. Evaporation rate of water without the membranes, the blank membrane and the membranes deposited with photothermal nanomaterials (TiO_2, TiON, TiN, carbon and graphene NPs) under simulated solar light with an illuminance of 550 W m^{-2}.

Material	Evaporation rate ($g\ min^{-1}$)	Equivalent evaporation rate ($kg\ h^{-1}\ m^{-2}$)
Water-only (no membrane)	0.018	0.55
Blank membrane	0.020	0.61
TiO_2 membrane	0.021	0.64
TiON membrane	0.032	0.98
TiN membrane	0.045	1.38
Nanocarbon membrane	0.032	0.98
Graphene membrane	0.033	1.01

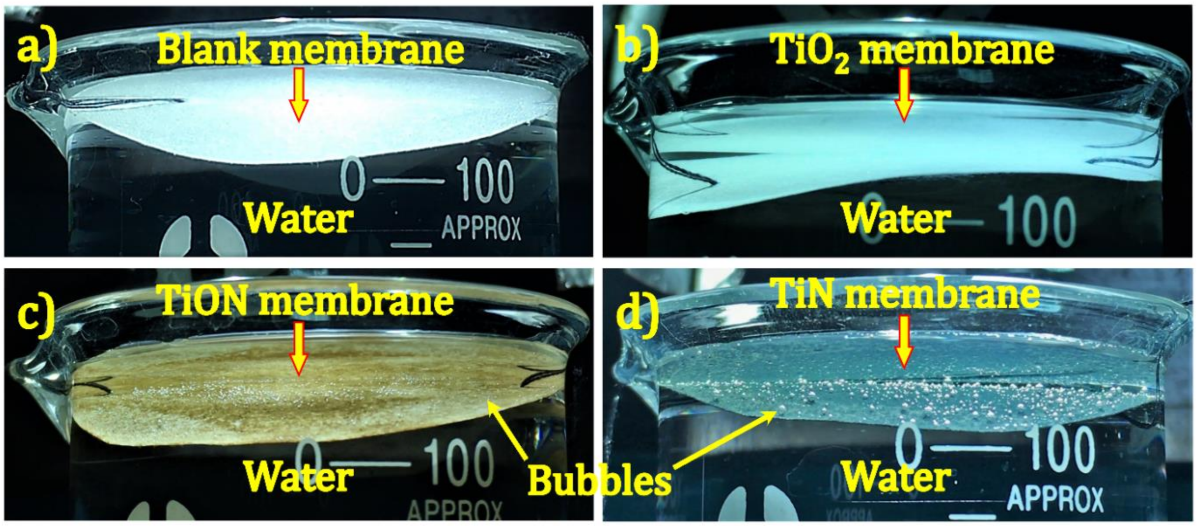

Figure S4. Photographs of (a) blank polymer membrane, (b) TiO_2 membrane, (c) TiON membrane and (d) TiN membrane taken after 600 s of exposure to simulated solar light generated by the Xenon arc lamp with an illuminance of 550 W m^{-2}. The membranes were slightly tilted for a better observation on the bubbles. No bubbles were observed for the blank and TiO_2 membranes, whereas sporadic bubbles appeared on the TiON membrane surface.

Video S1: Formation of bubbles on TiN membrane under continuous illumination of simulated solar light with an illumination of 550 W m^{-2}. The video was shortened by increasing the speed to 12 times (time lapse of 12x) of the normal speed.

Video S2: Evaporation of water in the presence of the TiN membrane under continuous illumination of simulated solar light with an illumination of 550 W m^{-2}.
